# Fantastic animals as an experimental model to teach animal adaptation

**DOI:** 10.1186/1471-2148-7-S2-S13

**Published:** 2007-08-16

**Authors:** Roberto Guidetti, Laura Baraldi, Caterina Calzolai, Lorenza Pini, Paola Veronesi, Aurora Pederzoli

**Affiliations:** 1 Scuola di Specializzazione per l'Insegnamento Secondario University of Modena and Reggio Emilia, Modena, Italy; 2 Scuola Media Statale "G. Marconi", Modena, Italy

## Abstract

**Background:**

Science curricula and teachers should emphasize evolution in a manner commensurate with its importance as a unifying concept in science. The concept of adaptation represents a first step to understand the results of natural selection. We settled an experimental project of alternative didactic to improve knowledge of organism adaptation. Students were involved and stimulated in learning processes by creative activities. To set adaptation in a historic frame, fossil records as evidence of past life and evolution were considered.

**Results:**

The experimental project is schematized in nine phases: review of previous knowledge; lesson on fossils; lesson on fantastic animals; planning an imaginary world; creation of an imaginary animal; revision of the imaginary animals; adaptations of real animals; adaptations of fossil animals; and public exposition. A rubric to evaluate the student's performances is reported. The project involved professors and students of the University of Modena and Reggio Emilia and of the "G. Marconi" Secondary School of First Degree (Modena, Italy).

**Conclusion:**

The educational objectives of the project are in line with the National Indications of the Italian Ministry of Public Instruction: knowledge of the characteristics of living beings, the meanings of the term "adaptation", the meaning of fossils, the definition of ecosystem, and the particularity of the different biomes. At the end of the project, students will be able to grasp particular adaptations of real organisms and to deduce information about the environment in which the organism evolved. This project allows students to review previous knowledge and to form their personalities.

## Background

It is impossible that any abstraction can form a subject of natural science, seeing that everything that Nature makes is means to an end.

"*On the Parts of Animals*" Aristotle

Science curricula and teachers should emphasize evolution in a manner commensurate with its importance as a unifying concept in science and its overall explanatory power. It provides students with powerful ideas to help them understand the natural world [1]. Scientific interpretations of natural events need interpretation and elaboration efforts that are rarely spontaneous; to lead students to their knowledge, comprehension and conceptualization is a demanding and exciting task for a teacher [2]. Evolutionary processes and, in general, scientific explanations of the world are often in contrast with the immediate and simple explanations that our brain gives of reality (e.g. the sun seems to turn around the earth, the earth seems to be flat), and are influenced by what Francis Bacon called "*idola*" (false notions or tendencies which distort the truth [3]). Due to the slowness of evolutionary processes, species seem immutable and fixed in time. Even though transformation and evolution are accepted as theoretical concepts, people often have a finalistic view of the evolutionary processes. This leads to the idea that: (a) organisms are perfectly adapted to their environment, (b) their characteristics cannot be other than they are, and (c) everything is made for the best purpose. These misconceptions are well represented by Voltaire through the words of Dr. Pangloss: "...the nose was created for the purpose of wearing spectacles. Legs were clearly intended for breeches, and we wear them" [4]. The Panglossian paradigm is a term coined by Gould and Lewontin [5] to refer to the notion that everything has specifically adapted to suit specific purposes.

Adaptation is a crucial concept of the evolutionary theory. Ridley in his recent book on evolution reports that "It is one of the main aims of modern evolutionary biology to explain the forms of adaptation that we find in the living world. Adaptation refers to those properties of living things that enable them to survive and reproduce in nature" [6]. For a better understanding of evolution, the concept of adaptation represents a first step to understand the results of natural selection.

We began our planning of this experimental project on the assumption that the "forms" of most characters (morphological, ultrastructural, molecular) of living beings are related to their functions, which in their turn are linked to the environments inhabited by organisms. While this is not always true, students need to understand that survival and fitness are dependent on structures and their functions. In this way they will come to understand the concept of natural selection [7].

The classic pedagogical approach, mainly based on frontal lessons supported by book reading, unlikely produces a constructivist view of knowing in students, but empirical experiences cannot always be used to understand particular concepts such as evolutionary theory. With this in mind, we developed an experimental project of alternative didactics to better understand previous disciplinary information (life science) and to improve knowledge of organism adaptation with an evolutionary perspective. We used inductive methods to direct student understanding about the concept of adaptation. The students were involved and stimulated in learning processes by creative activities, by laboratory work, by visits at different institutions, and by cooperative learning teaching structures. This experience was significant because it involved people (university professors, school teacher and their students) and institutions (university, school and museum) at different levels of teaching research.

Creative activities and fantastic animals were used, not only as a way to teach, but (also) as a way to capture students' interest and to keep it active during the project. To set adaptation in a historic frame, we also considered fossil records as evidence of past life and of evolution.

## Results and conclusion

Phases of the project (1–9) and their realization with the students (1a-9a):

### 1) Review of previous knowledge

Review or introduction to animal characteristics (review process). Prerequisites needed by students to follow and perform this teaching project are related to the definition of living systems and to the characteristics of the organisms (with emphasis on animals), such as life cycle, relationships between animal form and functions, and the main properties and characteristics of animals.

#### 1a) Realization

For the students, it was a review of previous concepts. This phase was performed in the classroom by frontal lessons and Think Pair Share as a cooperative discussion strategy [8]. This strategy is useful because it structures the discussion: (a) the teacher stimulates students with a question, prompt, or observation, using designated partners; (b) students talk about the answer each one has formulated and identify the best answers; and (c) then the teacher calls for pairs to share their thought processes with the rest of the class.

### 2) Lesson on fossils

A learning process is given to instruct students about the meaning of fossils as evidence of past life. There is a presentation on the different kind of fossil records and main processes leading to their formation. This learning process should be followed by a hands-on activity related to fossilization processes. In this phase the student should also understand the rarity of the event of fossilization.

Fantastic animals may have been introduced prior to this phase because fossils have been interpreted as traces or remains of monstrous creatures. For example, fossils of Belemnite shells were believed to be either remains of thunderbolt tips within the rocks or devil nails with curative power; Ammonite shells were considered remains of snakes; the myth of Cyclops probably originated from the discovery, in an ancient age, of fossils of draft elephants; fossils of giant salamanders were considered remains of a human who died during the universal flood. In addition, dinosaurs are popular extinct animals that always arouse interest in students.

#### 2a) Realization

This phase was conducted as a frontal lesson (in classroom) followed by a laboratory activity (in school laboratory), in which students participated in some simulated sedimentary processes with actual casts of shells and leaves they collected and brought to school.

### 3) Lesson on fantastic animals

Presentation of an overview of imaginary creatures, animals, and myths created throughout human history (stimulating process). This phase stimulates student curiosity and interest for the next steps of the project. Dealing with this topic gives the opportunity either to explain the characteristics of real animals cited in this exercise or to compare characteristics of fantastic animals with those of real animals.

This overview can be shown with different approaches and topics depending on the teacher and/or students' interests. With examples, we cite different possible approaches:

i. overview of the most famous imaginary creatures and their characteristics, with information of their origin [9];

ii. fantastic creatures that inspired scientific names (e.g. *Hydra *is a genus name of order Hydroida, *Cyclops *is a genus of subclass Copepoda; Family Sirenidae is a family of salamanders and Sirenia, an order of mammals; *Proteus *is a genus of amphibians; echidna is the common name of a mammal in order Monotremata; *Chimaera *is a genus of fish; and medusae are forms of cnidarians or a species name of an orchid);

iii. fantastic animals in science fiction, card games, and videogames. This approach should create an empathy with the world of fantasy associated to student everyday life (e.g. it is possible to study the life cycle and adaptations of Ridley Scott's "Alien" [10], Pokemon and Digimon cards can be used to analyze and compare environmental adaptations and evolution of characters, analysis of role-playing game characters may be helpful to identify parts of real animals used to create them);

iv. analysis of hybrid animal-plants (e.g. Barometz, Bernacae), human-animals (e.g. Centaur, Minotaur, Siren) or human-plants (e.g. wak-wak, Mandragora);

v. creatures between science and science fiction studied by cryptozoologist [11] as "Nessie" the Monster of Loch Ness [12,13], the Yeti, the Bigfoot and the Sasquatch [14,15]. The interesting case of the Piltdown man (*Homo piltdowni*), the 'missing link' between apes and humans, was found to be a hoax [16-18]. This topic can also be useful to explain what "science" is, what the scientific method is, and how science knowledge is derived from the accumulation of data;

vi. introduction to the Renaissance "Wonderkammer" and an overview of the stunning adaptations produced by the process of natural selection in real animals, showing the natural world as a huge "Wonderkammer". Examples of abyssal creatures, the organization of social insects, mimetic adaptations, and organisms with strange features can be use to show that "the zoology of the dreams is poorer than the zoology of God" [9].

#### 3a) Realization

This phase was performed as a conference on fantastic animals with the title "The Monstrous and the Fantastic: A Journey in the Zoology between Myth and Reality", a time travel from prehistory to the present in which the fantasy of the human being and the reality of nature intertwine and compete in the creation of creatures whose power of suggestion continues today. The conference was held at the university by a university zoologist. The conference, held in a university classroom, gave the opportunity for students to meet the "university world", of which they frequently hear but can rarely explore.

### 4) Planning an imaginary world/environment

Planning an imaginary world/environment (creative process) has to be explained to students that the world/environment to be created is to have an intrinsic coherence (e.g. without any source of heat, such as energy from stars or volcanoes; the temperature can not be high; and low gravity does not allow dense atmosphere, etc.). After the creation of the imaginary cosmos, its characteristics have to be discussed with students, identifying possible incongruence or excessively fantastic characteristics. The properties of the imaginary world/environment have to be adjusted or corrected according to results of the discussion. This led the students to review previous knowledge on several topics related to subjects such as geology, chemistry, physic, geography, and botany.

As an alternative option, the teacher can plan and create imaginary worlds/environments to be assigned to students [19], so that the teacher can manage each step of progress.

#### 4a) Realization

**Figure 1:**
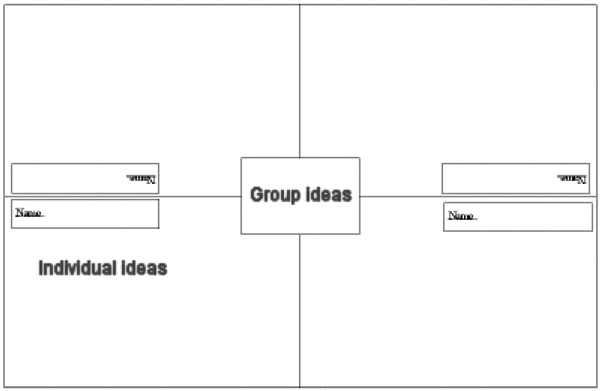
**Scheme of the poster used for the cooperative learning strategy "Consensus Placemat"**. When using this tool, students work individually to brainstorm their ideas about a topic (Individual ideas) and then combine their most important ideas with ideas of others in their group (Group ideas). (Picture and text from: ).

This phase was performed in classroom with the help of the teacher using a cooperative learning strategy called the Consensus Placemat [20,21] (Figure 1). Placemat allows students to think about, record, and share their ideas around a key idea or issue. When using this tool, students work individually to brainstorm their ideas about a topic and then combine their most important ideas with ideas of others in their group. Members of the group discuss the ideas selected by individual members and, with a consensus, develop a group list. At the end of this work the students produced posters with a drawing (usually a map) and a list of characteristics (e.g. temperature, day length, water availability, gravity, composition of atmosphere, mountains, vegetation) of the imaginary world/environment. Posters were hung in the classroom, and then students proceeded with a gallery tour [20,21] to view and discuss posters of other groups. After the gallery tour, the teacher proceeded with the plenary discussion of the world/environment created by students so that they could perform any changes that might be needed.

### 5) Creation of an imaginary animal adapted to the imaginary world/environment

**Figure 2:**
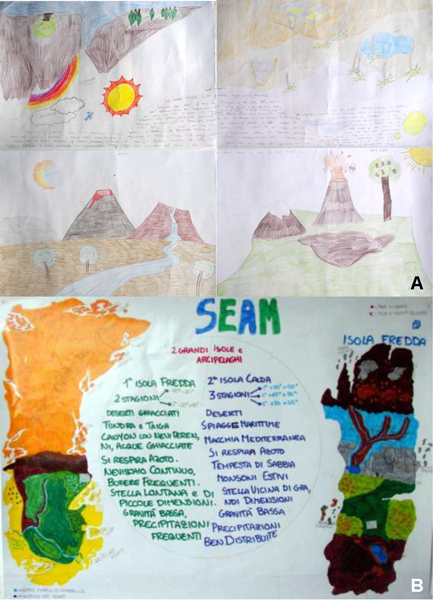
**Examples of posters realized by groups of students using the "Consensus Placemat" strategy**. Students produced posters of the imaginary world/environment (A, B) by drawing a map and a list of characteristics (e.g. temperature, day length, water availability, gravity, atmosphere composition, mountains, vegetation) of the imaginary world/environment. Posters were hung in the classroom for the gallery tour. A. The "Wild World" planet is characterized by mountains (covering the 75% of planet), rivers and active volcanoes. The climate is temperate. B. The "SEAM" planet has low gravity and it is characterized by two large islands with almost opposite environmental characteristics. The "Cold Island" with ice desert, tundra and taiga; the "Warm Island" with mountains and sandy dunes, covered by Mediterranean macchia-grassland.

Task for students: creation of an imaginary animal adapted to the imaginary world/environment (creative process) (Figure 2). Each student created her/his own animal giving it a scientific name (according to the International Code of Zoological Nomenclature [22]), describing and drawing its life cycle, morphology, and anatomy.

This led students to review knowledge of animal morphology and anatomy, relationships between form and function, and adaptations of organisms to their environment. The student learned how: (a) a scientific name is attributed to a species after its discovery and (b) the rules of the scientific community for such topic.

#### 5a) Realization

**Figure 3:**
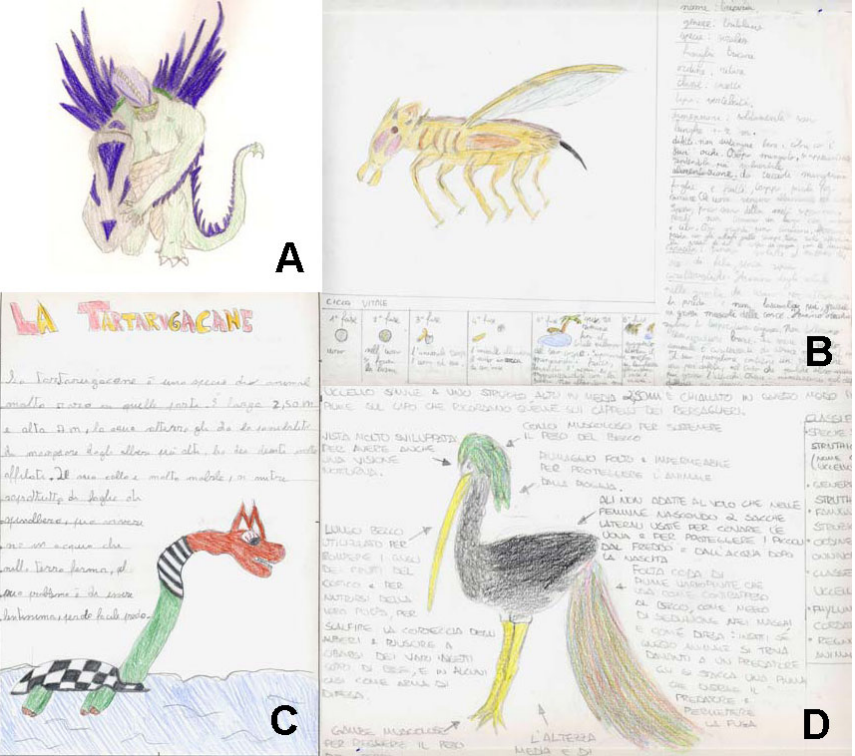
**Examples of some imaginary animals created by students**. A. *Scutum squamatum*. Habitat: cold and wet areas (Cold Island of SEAM planet; Fig. 2B). Characteristics: bone-shield on a leg, large claws and dorsal spines to capture preys and to protect its hunting territory, a layer of fat under the scaly skin to protect from cold and as energy supply. B. *Tritolaus siralis*. Habitat: dry and hot areas (Warm Island of SEAM planet; Fig. 2B). Characteristics: cryptic color, ears for sound perception and thermoregulation, claws and jaws to capture and eat prey. C. *Tartarus canis*. Habitat: land and freshwater (Wild World; Fig. 2A). Characteristics: very long neck to reach tree leaves, flipper-legs to crawl and swim. D. *Struthio becchi*. Habitat: temperate areas (Wild World; Fig. 2A). Characteristics: large eggs carried in lateral bags under the wings, long beak to break seeds and to bark trees looking for insects, tail feathers to attract partners and for defence (a feather can be removed to disorientate a predator).

In the classroom, the teacher explained the task and presented basic rules of the International Code of Zoological Nomenclature. Students, individually, performed the task for homework (Figure 3).

An important goal of this step was the spontaneous creation, by the student, of trophic relationships among the imaginary animals of a same world/environment, although this was not specifically requested in the task.

### 6) Revision and discussion of the imaginary animals

Two aspects have to be analyzed and discussed with the students: (a) the congruence among the animal characteristics and (b) the congruence between the animal adaptations and its environment (analytical process). After this analysis, students have to correct their animals according to results of the discussion.

To judge the student's work, a rubric such as that presented in Table 1, can be used.

**Table 1:** Rubric to judge the student's work.

	Level 1	Level 2	Level 3
	EXCELLENT/VERY GOOD	GOOD/PASS	FAIL

Presence of adaption requirements to the environment	Adaptations are coherent and described in a detailed way. The fantastic animal shows almost all the adaptive elements to survive and complete the life cycle	The fantastic animal shows a sufficient amount of adaptive elements to complete its life cycle, even if it is not described in a detailed way	The fantastic animal shows no or only few adaptive elements, those present not always being coherent
Completeness respect to the task requests	All animal characteristics are considered, the description is complete and coherent	Only some animal characteristics are considered, the description is superficial	Only few animal characteristics are considered, the description is incomplete or incorrect
Coherence between drawing and description	The drawing is rich of details and reflects the animal description	The drawing has few details and reflects only partially the animal description	The drawing does not reflect the animal description or is absent
Coherence among information	Information is coherent with the animal and environment descriptions	Information is quite coherent with the animal and environment descriptions	Information is not or not very coherent with the animal and environment descriptions

#### 6a) Realization

In the school classroom, an open and plenary discussion [23] among the students, the teacher, and a university zoologist were useful in analyzing the animals created by students.

### 7) Identification and discussion of the adaptations of real animals

This phase is important because it represents the synthesis activity of previous steps. After understanding relationships between animal form and function, and between function and environment, the student should be able to: (a) grasp specific adaptations of real organisms and (b) deduce information on the environment in which they evolved (synthesis process). This phase can be accomplished either: (a) by a visit to a Zoological Museum, (b) in the classroom using pictures of real animals, or (c) in the laboratory observing animals (or part of them) collected by the students.

It would also be important to perform this analysis work in relation to characteristics of the human being, to understand why human beings, in specific geographic locales, evolved with determinate features. For example, why people have different skin colors, why various eye and nose shapes, why each one of us is different from everyone else, why human males and females are different, and so forth.

Wrong interpretations by scientists or applications of "common sense" about the origins of some human features can be used in these teaching activities. For example, questions such as why we resemble a "Naked Ape" or why both men and women have nipples. These questions were addressed with the following incorrect answers: the reduction of hairs in humans is due to an aquatic evolutionary phase of human beings that selected for a reduction of hairs to reduce the water friction [24,25]; men have nipples due to a past role of their suckling newborns [26].

#### 7a) Realization

This phase was developed within a guided tour in the Zoological Museum of the University of Modena and Reggio Emilia, with the aim of discovering and discussing adaptations of real animals. Students were intrigued in the work of an expert museum guide who stimulated them to: (a) find out the animals' environments, (b) observe their feeding habits, and (c) see how these habits compared to the animal's morphology.

### 8) Identification and discussion of fossil animals' adaptations

Students conduct identification of morphology and autoecology of extinct organisms by analysis of fossil records (synthesis process). Students have to be stimulated to perform the same work described in step 7 but this time with fossil animals. For example, paleontological records can be viewed in Paleontological Museums, in the classroom with pictures, and/or by analyzing students' fossil finds. After completing this task, students should be able to understand that the modern and ancient animals and communities are/were adapted to their environments and that the natural forces that create/created those adaptations are/were the same in both cases. Also, students should (a) evaluate the importance of fossils for our knowledge of past life, (b) identify conditions necessary for fossilization, (c) and construct a possible scenario for the formation of fossils [27].

#### 8a) Realization

This step was impossible to perform with students.

### 9) Public exposition of the experimental project

This is another important step of the work because it allows students to review all their work, to reflect upon and synthesize their new knowledge (metacognitive process). Moreover, it allows them to attain objectives related to the formation of their personalities because students are stimulated to use an appropriate language, to test their exposition capabilities, and to overcome their shyness.

This step can also represent a way for the teacher to verify and judge a student's final works.

#### 9a) Realization

The students present their works to the public of the Zoological Museum during the International Museum Day ("Museums and young people" 18 May 2006). The museum's current theme was to allow the museum community to reflect upon, address, and celebrate the role young people play in fulfilling their mission, and to sensitize the public to their contribution toward a more solidarious and tolerant society [28].

## Methods

This project was planned and performed during the years 2005/2006. It has involved professors and students of the School of Specialization for the Secondary Teaching (SSST) of the University of Modena and Reggio Emilia (Modena, Italy), a teacher and students of the Secondary School of First Degree "G. Marconi" (Modena, Italy). The School of Specialization is a two-year course for graduate students wanting to take up a career as teachers in the Italian Secondary School of First Degree (student age 11–14 years). The institutions involved in the project were the University of Modena and Reggio Emilia, the University Museum of Zoology and Comparative Anatomy and the Secondary School of First Degree "G. Marconi".

The project has been developed during the SSST course. The SSST students experienced all the project phases, understanding the impact on the young students of each project phase and of each cooperative learning structure that we used. In this way it was possible to apply a backward designed strategy [29] to review each phase for a better efficiency of the whole project. Then the project was proposed to a teacher of the "G. Marconi" School, who agreed to perform it with her students (aged 13–14). The project was introduced during the optional curriculum of science laboratory, and it was completed in about 30 hours. Students were so enthusiastic about the project that they dedicated time to it beyond the ordinary school's scheduled time.

The experimental project we are presenting can be viewed as a framework, whose phases can either be developed or be reduced in accordance with the available time for the activity and/or with specific topics not explicitly treated in this paper. Imaginary animals created by the students can be also used as a tool within the entire school curriculum. If the student creates an imaginary animal at the beginning of the classes, she/he could add any biological information to this imaginary creature that she/he learns during the science courses. This would allow a recurrence in teaching practice, a kind of spiral curriculum [30] to reinforce and enrich concepts, thus increasing the cognitive conceptual map of students.

The project has the following educational objectives: (a) knowledge the characteristics of living beings (life cycle, morphology, anatomy, physiology), (b) meanings of the term "adaptation" in biology, (c) meaning of fossils and the fossilization processes, (d) definition of ecosystem, and (e) particularity of the different biomes.

At the end of the project, the student should be able to: (a) correlate the main functions of a living being (feeding, respiration, excretion, transport, perception, etc.), (b) know an ecosystem and the factors and conditions for its equilibrium, (c) find the relationships between organisms and their environment, (d) find the relationships among organisms of an ecosystem, (e) understand that to survive and reproduce an organism has to be adapted to its environment, and (f) understand the importance of fossils in the development of evolutionary theories.

Other teaching outcomes of this project are related to formation of student personality. The student activities that we propose require interaction among intelligence, understanding, interpretation, imagination, and creativity. Moreover, students have to (a) interact among themselves and among people from another school world, (b) find common and shared solutions, and (c) live the school as a place of creativity and not just academics.

The objectives of this project are in line with the National Indications of the Italian Ministry of Public Instruction. Specific competencies are required for students: observation of the reality to recognize relationships, modifications, and causal connections; understanding of the typical elements of the natural and anthropic environments; and development of study and research attitudes on natural world [31]. Moreover, this project is useful as an introduction to other required scientific competencies: knowledge of and reflection upon evolution of living beings and Darwinian theories.

The experimental project can be schematized in nine phases (developed and discussed in Results and Conclusions session):

1) Review of previous knowledge (review process).

2) Lesson on fossils (learning process).

3) Lesson on fantastic animals (stimulating process).

4) Planning an imaginary world/environment (creative process).

5) Creation of an imaginary animal adapted to the imaginary world/environment (creative process).

6) Revision and discussion of the imaginary animals (analytical process).

7) Identification and discussion of the adaptations of real animals (synthesis process).

8) Identification and discussion of the adaptations of fossil animals (synthesis process).

9) Public exposition of the experimental project (metacognitive process).

This experience can also be adapted as a good interdisciplinary project that can involve scientific topics such as biology, geology, physics, chemistry, and humanistic topics such as geography, literature, and history. This project can also be adapted for high school students. In this case an inspiring reading could be "The Snouters: Form and Life of the Rhinogrades" [32], in which an imaginary explorer describes, in a scientific way, a series of fantastic creatures, their adaptations, and habitats of islands from which they evolved.

We asked SSST students to create a rubric to evaluate the student's performances of this teaching project. A rubric is a scoring guide to judge the student's work based on the sum of a full range of criteria, rather than a single numerical score [33]. It represents a working guide for the teacher but it can be handed out to students before the assignment begins in order to make them familiar with the criteria on which their work will be judged. In this way students understand how they will be evaluated and can prepare accordingly, enhancing the quality of direct instruction.

This teaching project was presented at the 2^nd ^Meeting of Italian Evolutionary Biologists – 1^st ^ISEB Congress (Florence, Italy, 2006) and it won *ex aequo*, the prize as best poster of the congress [34].

## Competing interests

The authors declare that they have no competing interests.

## Authors' contributions

RG and AP authors and supervisors of the project. LB museum guide and supervisor of the student's public exposition. CC school teacher and supervisor of the classroom activities and public exposition. LP and PV advisers and supervisors of teaching project and cooperative learning strategies. All authors read and approved the final manuscript.
